# Community intervention for child tuberculosis active contact investigation and management: study protocol for a parallel cluster randomized controlled trial

**DOI:** 10.1186/s13063-021-05124-9

**Published:** 2021-03-02

**Authors:** Anca Vasiliu, Sabrina Eymard-Duvernay, Boris Tchounga, Daniel Atwine, Elisabete de Carvalho, Sayouba Ouedraogo, Michael Kakinda, Patrice Tchendjou, Stavia Turyahabwe, Albert Kuate Kuate, Georges Tiendrebeogo, Peter J. Dodd, Stephen M. Graham, Jennifer Cohn, Martina Casenghi, Maryline Bonnet

**Affiliations:** 1 French National Research Institute for Sustainable Development (IRD UMI 233 TransVIHMI- UM-INSERM U1175), Montpellier, France; 2 Elizabeth Glaser Pediatric AIDS Foundation, Yaoundé, Cameroon; 3 Epicentre Research Center, Mbarara, Uganda; 4 Elizabeth Glaser Pediatric AIDS Foundation, Mbarara, Uganda; 5 National Tuberculosis and Leprosy Program, Kampala, Uganda; 6 National Tuberculosis Program, Yaoundé, Cameroon; 7 School of Health and Related Research, University of Sheffield, Sheffield, UK; 8 Centre for International Child Health, University of Melbourne and Murdoch Children’s Research Institute, Royal Children’s Hospital, Melbourne, Australia; 9 International Union Against Tuberculosis and Lung Disease (The Union), Paris, France; 10 Elizabeth Glaser Pediatric AIDS Foundation, Geneva, Switzerland

**Keywords:** Contact tracing, Preventive therapy, Pediatric tuberculosis, Community intervention, Tuberculosis symptom screening, Cluster randomized controlled trial

## Abstract

**Background:**

There are major gaps in the management of pediatric tuberculosis (TB) contact investigation for rapid identification of active tuberculosis and initiation of preventive therapy. This study aims to evaluate the impact of a community-based intervention as compared to facility-based model for the management of children in contact with bacteriologically confirmed pulmonary TB adults in low-resource high-burden settings.

**Methods/design:**

This multicenter parallel open-label cluster randomized controlled trial is composed of three phases: I, baseline phase in which retrospective data are collected, quality of data recording in facility registers is checked, and expected acceptability and feasibility of the intervention is assessed; II, intervention phase with enrolment of index cases and contact cases in either facility- or community-based models; and III, explanatory phase including endpoint data analysis, cost-effectiveness analysis, and post-intervention acceptability assessment by healthcare providers and beneficiaries. The study uses both quantitative and qualitative analysis methods. The community-based intervention includes identification and screening of all household contacts, referral of contacts with TB-suggestive symptoms to the facility for investigation, and household initiation of preventive therapy with follow-up of eligible child contacts by community healthcare workers, i.e., all young (< 5 years) child contacts or older (5–14 years) child contacts living with HIV, and with no evidence of TB disease. Twenty clusters representing TB diagnostic and treatment facilities with their catchment areas are randomized in a 1:1 ratio to either the community-based intervention arm or the facility-based standard of care arm in Cameroon and Uganda. Randomization was stratified by country and constrained on the number of index cases per cluster. The primary endpoint is the proportion of eligible child contacts who initiate and complete the preventive therapy. The sample size is of 1500 child contacts to identify a 10% difference between the arms with the assumption that 60% of children will complete the preventive therapy in the standard of care arm.

**Discussion:**

This study will provide evidence of the impact of a community-based intervention on household child contact screening and management of TB preventive therapy in order to improve care and prevention of childhood TB in low-resource high-burden settings.

**Trial registration:**

ClinicalTrials.gov NCT03832023. Registered on 6 February 2019

**Supplementary Information:**

The online version contains supplementary material available at 10.1186/s13063-021-05124-9.

## Background

Tuberculosis (TB) is an infectious disease causing incident cases of disease in around 10 million people worldwide in 2018 [1]. The World Health Organization (WHO) estimated that 11% of the TB cases in 2018 were in children (< 15 years). However, a modeling study has estimated that the pediatric caseload in high-burden countries is as high as 15–20% of all TB cases [2]. The mortality rate in undetected untreated children is estimated to be 21.9% for children of all ages and rises to 43.6% in young children of less than 5 years [3]. Improving case detection and treatment of this high-risk group of young children is particularly challenging due to diagnostic limitations and clinical overlap with other common severe diseases of infants and young children in resource-limited settings.

Research has consistently shown that TB disease in young children usually occurs soon after exposure and infection, that the risk of disease if infected is high, and that TB preventive therapy (TPT) can significantly reduce the risk of disease following exposure and infection [4]. A meta-analysis reported that 10% of young child contacts had TB disease at the time of screening, and 35% had evidence of infection [5]. A recent individualized participant meta-analysis found that the effectiveness of tuberculosis preventive treatment (TPT) was 63% (95% CI 53–70%) among all exposed children and 85% (95% CI 80–89%) among those with evidence of infection [4]. Therefore, the rapid identification and management of exposed children in the households of TB disease cases is a critical opportunity to detect, treat, and prevent TB. Although recommended for decades, household child contact screening and TPT have been poorly implemented in high-burden and resource-limited countries. For many years, children were considered lower priority due to being less infectious and therefore contributing less to TB transmission than adults [6].

The WHO End TB Strategy and ambitious targets for coverage of screening and TPT in the Global Plan to End TB demonstrate political will and provide renewed opportunity to close the wide policy-practice gap [7, 8]. The policy-practice gaps observed in the screening and management of child contacts are driven by health system and human resource challenges as well as the many challenges faced by families in bringing their children to the health facility [9–13]. In most low-resource countries, the index case is asked to bring all child contacts to the health facility for TB screening, and yet many barriers arise when applying this recommendation such as scheduling or financial challenges, transport costs, long waiting periods in settings with risks of further exposure, and families or even healthcare workers’ reluctance to apply these guidelines as they do not always understand the rationale, potential benefits, or risks when the child is well [13–17]. Community-based household contact screening of children in the household is likely to improve TB disease case detection [8, 18–20].

The use of classical tuberculin skin test (TST) to identify child contact with TB infection who will benefit from the TPT and the need of chest radiography (CXR) in addition to symptom screening to exclude TB disease before initiation of TPT have both operational challenge that contributes to the lost proportion of child contacts initiated on TPT for a long time [13]. However, there is evidence that the additional yield of TB disease detection from CXR in asymptomatic child contacts is extremely low [9, 21–23]. In addition, WHO has recommended since 2006 that high-risk child TB contacts—young (< 5 years) or are living with human immunodeficiency virus (HIV) of any age—receive TPT after exclusion of TB disease without systematically confirming TB infection with TST [24, 25]. Therefore, a symptom-based approach that does not require further investigations for asymptomatic child contacts could facilitate a more decentralized, community-based implementation to initiate TPT in asymptomatic children [8]. In addition, the recent WHO recommendations [6] that include shorter TPT combination regimens (isoniazid and rifampicin or rifapentine for 3 months) are associated with improved adherence compared to the standard TPT regimen of isoniazid monotherapy for at least 6 months and provide an important opportunity for increasing completion of TPT [9, 26, 27]. Further, follow-up of children receiving TPT at the household could further improve TPT completion rates and could be easily integrated with activities to support treatment of TB disease of the index cases in the household.

There is no published study that has evaluated the impact on the cascade of care of pediatric TB case detection and preventive therapy management of a community-based approach compared to a facility-based standard of care. We therefore aim to evaluate the implementation of a community-based approach to child TB contact screening and management in two TB-endemic African countries.

## Methods

### Study objective and endpoints

The primary objective of this study is to compare the proportion of household child TB contacts eligible for TPT who initiate and complete TPT under a facility-based standard of care and under a decentralized community-based intervention model of care for contact screening and management.

The corresponding primary endpoint is the proportion of child TB contacts < 5 years of age and HIV-infected children of 5–14 years of age who are declared by the index case and who initiate and complete the TPT.

The secondary objectives compare the aforementioned models in terms of (i) cascade of care of TPT initiation and completion in child contacts < 5 years or HIV-positive children 5–14 years; (ii) cascade of care for TB detection and treatment in all included contacts; (iii) tolerability and adherence in children initiated on TPT; (iv) acceptability and feasibility of the two models by the parents/guardians, health personnel, and community; (v) the effect of the community-based intervention on the number of adult contacts diagnosed with TB; and the cost-effectiveness. The number of children and adults diagnosed with TB and the number of children initiated on TPT will be also compared before and after the intervention.

The secondary endpoints of the study are presented in Table 1.

**Table 1:** Secondary endpoints of the CONTACT study

General secondary endpoints	Detailed secondary endpoints
Cascade of care for the initiation and completion of TPT of child contacts < 5 years or HIV-infected 5–14 years and reasons of dropouts at different steps of the cascade	Number of screened children and proportion of children screened among child contacts < 5 years or HIV-infected 5–14 years declared by the index case
	Proportion of children potentially eligible for TPT: TB disease excluded
	Proportion of children eligible for TPT after exclusion of contraindication to TPT
	Proportion of children started on TPT among those eligible for TPT
	Proportion of children who did not complete TPT among those started on TPT and reasons of interruptions
Cascade of care for TB detection of child contacts	Proportion of children with symptoms suggestive of TB: presumptive TB
	Proportion of presumptive TB cases investigated for TB
	Proportion of children diagnosed with TB
	Proportion of children with TB diagnosis who are started on TB treatment
Cascade of care for TB detection of adult contacts	Number of adults screened and proportion of adults screened among household identified adult contacts
	Proportion of adults with symptoms suggestive of TB: presumptive TB cases
	Proportion of adults presumptive TB cases diagnosed with TB
Safety and treatment adherence for children under TPT	Proportion of children with serious adverse events
	Proportion of children with adverse events of interest: peripheral neuropathy, clinical hepatotoxicity
	Ratio of dose taken as indicated (ticked) on the treatment card by the parent/guardian over the total number of doses to be taken by prescription
Endpoints at 6 months	Treatment outcomes of children started on TB treatment
	Proportion of children diagnosed with TB after initiation of TPT: during and after TPT
	Proportion of children diagnosed with TB among those who were not started on TPT and were not diagnosed with TB at baseline assessment
Before-after comparison for TB adult and pediatric cases and for TPT initiation and completion from health facility registers	Number of patients diagnosed with TB and registered
	Proportion of children among all patients diagnosed with TB and registered
	TB treatment outcomes of patients (adults and children) diagnosed with TB and registered
	Number of children started on TPT
	Completion rate of children started on TPT
Acceptability and feasibility of the intervention	Attitudes, willingness, and motivation to have a visit in their household
	Myths, anticipated fears, stigma, and risks of having a visit in their household
	Actual experiences with household visits
	Perception of the disease, its risk, and the notion of prevention, including TPT
	Description of critical events during house visits and how these were dealt with
	Identification of main constraints
Fidelity of the study	Proportion of delivered activities compared to the intended activities of the model
Cost-effectiveness of the intervention	Costs of CHW/community nurse assessment and treatment
	Costs of facility-based assessment and treatment
	Other facility costs (overheads, diagnosis, hospitalizations)
	Direct parent/guardian costs (related to care)
	Indirect parent/guardian costs (unrelated to care, e.g., dissaving, travel costs)

### Study design

This is a two-arm parallel cluster randomized study comparing two models of care for TB contact investigation and management. This study contains three phases:

1. Baseline phase (phase I) in which retrospective data collection and register quality checks were done in order to assess if the facility registers could be a reliable source of documents for the study. During this phase, there was also a baseline qualitative assessment with adult TB patients who are parents and stakeholders to better prepare the intervention phase and assess the acceptability and feasibility of the proposed activities.
2. Intervention phase (phase II) includes implementation and participant recruitment in the two models of care and study data collection.
3. Explanatory phase (phase III) contains the endpoint analysis and reporting, a cost-effectiveness analysis, and a post-intervention qualitative assessment with adult TB patients who are parents and cluster stakeholders to collect the acceptability of the implemented package.

This research is known under the name of CONTACT study (Community Intervention for Tuberculosis Active Contact Tracing and Preventive Therapy) and represents a research project embedded in a multi-country pediatric TB implementation program called Catalyzing Pediatric TB Innovations (CaP TB) led by the Elizabeth Glaser Pediatric AIDS Foundation (EGPAF) and funded by Unitaid.

### Study setting

This study is conducted in two high TB incidence, resource-limited African countries: Cameroon located in West Africa and Uganda in East Africa, with important differences in programmatic delivery of TB services. In Cameroon, TB care and management is centralized. Only secondary-level health facilities have TB laboratory diagnostic facilities and TB patients can only access care and drugs from these health facilities. In Uganda, TB management is decentralized to the primary healthcare level. In both countries, national guidelines [28, 29] at the time of this study development recommended contact investigation and screening as well as TPT with 6 months of daily isoniazid (6H) for eligible children. However, coverage of TPT for eligible children below 5 years is low in both countries. WHO recently reported that only 24% and 15% of eligible children were initiated on TPT in 2018 in Cameroon and Uganda respectively [1].

### Description of the intervention

#### Facility-based model

This model implements a “passive” approach to the screening and management of household contacts (see definition in the “Study population” section) at the facility level as per current practice. Implementation follows current National Tuberculosis Program (NTP) recommendations, except that a 3-month regimen of daily rifampicin-isoniazid (3RH) as a fixed-dose combination (FDC) is offered as TPT to eligible child contacts. In the context of the study, sites also benefit from additional data collection and trainings with follow-up support for the facility staff.

**Fig. 1:**
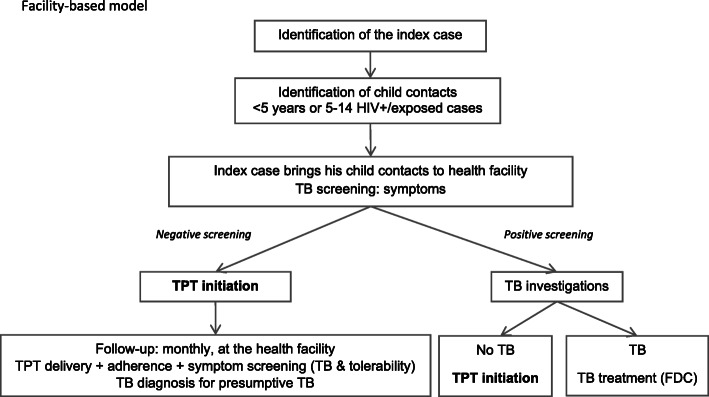
Facility-based model flowchart

When a person is diagnosed with bacteriologically confirmed pulmonary TB (index case), the facility staff in charge of TB (TB focal person) asks the index case to bring all household contacts with TB-related symptoms, all young (< 5 years) child contacts or older (5–14 years) child contacts living with HIV or exposed to HIV, irrespective of symptoms, to the health facility for evaluation for TB disease or for eligibility for TPT. This facility-based model is currently implemented in district hospitals in Cameroon for child contacts under 5 years old, but poorly applied for child contacts 5–14 living with HIV. In Uganda, the NTP allows household contact tracing when feasible but evaluation, TPT initiation, and follow-up are required to be done at the facility. In practice, due to lack of transport, the household contact tracing was poorly implemented. In both countries, at facility, TB investigations include clinical examination, sample collection for smear microscopy or Xpert MTB/RIF testing, and CXR when available and indicated, i.e., Xpert is negative or not done. Any contact diagnosed with TB is commenced on TB treatment, registered, and provided with treatment support and follow-up as per NTP guidelines. All sites are supported by the CaP TB program reducing the risk of heterogeneity of diagnosis and treatment of pediatric TB between sites and the two countries. Asymptomatic children who are eligible to receive TPT as 3RH (or 6H if drug-drug interactions with antiretroviral therapy preclude the use of rifampicin) are initiated at the facility with monthly follow-up (Fig. 1). The schedule of the facility-based model is presented in Table 2.

**Table 2:** SPIRIT study schedule

	Baseline phase		Intervention phase								Evaluation phase
	Allocation*	Site assessment**	Enrolment of index cases	Enrolment of contact cases	Follow-up					End of study	
**Timepoint**			**-2 W**	**0**	***W1***	***W2***	***W4***	***W8***	W12***	***W24***	
**Enrolment**											
**Eligibility screen**			X	X							
**Informed consent**			X	X							
**Allocation**	X										
**Interventions**											
**Facility-based arm**			X	X			X	X	X	X	
**Community-based arm**			X	X	X	X	X	X	X	X	
**Qualitative assessment**		X									X
**CEA**					X						
**Assessments**											
**Identification of contact children**			X								
**Children started on TPT**				X							
**Children completed TPT**									X	X	
**Acceptability of the intervention**		X									X
**TPT cost**					X						

*Abbreviations*: *W* week, *CEA* cost-effectiveness analysis, *TPT* TB preventive therapy

*Allocation takes place before the intervention begins as the clusters are randomized and not the individuals

**The baseline phase takes place 3 months before the intervention phase

***Children started on 6 months of isoniazid have 2 additional follow-up visits at week 16 and week 20

#### Community-based model

**Fig. 2:**
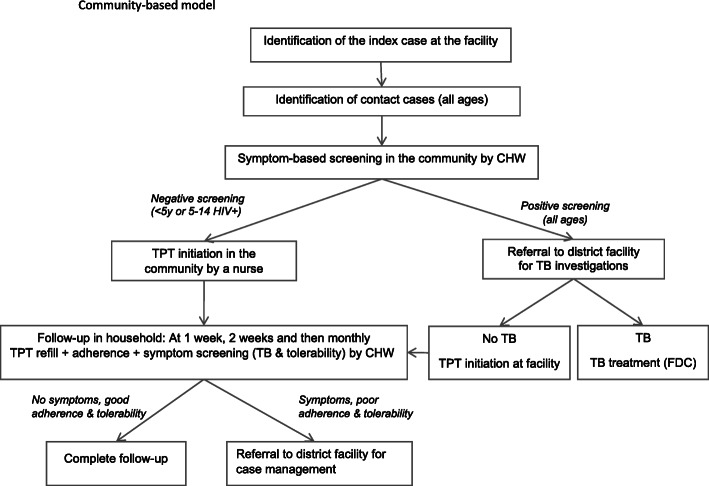
Community-based model flowchart

The intervention model is a decentralized, “active” approach to the screening and management of household contacts and is community-based. When an index case is diagnosed with bacteriologically confirmed pulmonary TB, the TB focal person asks whether s/he has child contacts in the household, and if so, then asks whether s/he is willing to receive a team in his/her household for contact symptom screening. If they agree, then an appointment is made and a team comprising a trained community health worker (CHW) and a research assistant goes in the household to screen all contacts (children and adults). If the index case does not have contact children in their household, then s/he is not included in the study, but contact investigation is done under routine care by the TB focal person. During the contact screening visit, the contacts who present symptoms of TB are referred to the health facility for TB investigations. Those who are asymptomatic and eligible for TPT (i.e., < 5 years irrespective of HIV status or 5–14 years and living with HIV) receive another visit by the TB focal person or TB nurse to initiate 3RH (or 6H if drug-drug interactions with antiretroviral therapy preclude the use of rifampicin). The follow-up is done at the household by the CHW after 1 week, 2 weeks, and then monthly in order to rapidly identify the children who develop TB symptoms in the community. The CHW collects the TPT at the health facility before each household visit and brings the remaining pills and documents back to the facility after the visit. During the follow-up visits, the CHW repeats the TB symptom screening, assesses the child’s TPT tolerability and adherence, and assesses the presence of any critical sign. If the child presents critical danger signs, tolerability problems, or TB symptoms, s/he is immediately referred to the health facility for a clinician to consult them (Fig. 2). The schedule of the community-based model is presented in Table 2.

### Study population

Bacteriologically confirmed (by smear microscopy, Xpert MTB/RIF, or TB loop-mediated isothermal amplification (TB-LAMP) assays) index cases, > 15 years old who have been diagnosed less than a month prior to inclusion and declaring child contacts in the study catchment area, are eligible. Exclusion criteria are known multi-drug resistance (MDR), the index case being a prisoner, or TB patients from an already screened household from the study.

Contacts sharing the same enclosed space for frequent or extended periods of time with the index case or having slept in the same bed during the last 3 months as per the WHO definition of a contact case [24] are eligible unless they are already on TPT or on TB treatment.

For the qualitative assessment, the study population is represented by key informants (facility managers, health staff, community health workers, and community leaders) and by male and female TB patients, who are parents/guardians.

### Cluster selection and randomization

The study clusters are health facilities supported by the CaP TB Program with TB diagnostic and treatment capacity after an initial assessment taking into consideration the number of bacteriological index cases identified from January to December 2018 (minimum of 50). Priority was given to rural, semi-rural, or semi-urban facilities as there is less population movement than in an urban setting with relatively easy access. In Cameroon, there were mainly district hospitals because TB diagnosis is mainly done at the secondary healthcare level, with ten clusters selected from two regions (Central and Littoral regions). In Uganda, as TB services are decentralized at the primary healthcare level, the ten clusters were primary health centers in four districts in the South West region, some with two facilities per cluster in order to reach the minimum of 50 index cases per year.

The randomization was stratified by country, and in each country, the 10 clusters have been allocated to one of the study models by a covariate-constrained randomization [30] taking into account the number of bacteriologically confirmed TB cases from that cluster the previous year. The randomization was performed by a statistician from the central research team 3 months prior to the start of inclusions. Participants, healthcare providers, study staff, and investigators are not blinded to the allocation of the health facilities.

The cluster list can be found in the Supplementary Material.

Criteria to discontinue the allocated intervention to a cluster are the absence of recruitment in a cluster for more than 2 months or if the NTP proposes a similar intervention that would bias the outcomes of the study.

### TPT

The 3RH regimen uses the child-friendly formulation of rifampicin (R) 75 mg/isoniazid (H) 50 mg as a FDC [31] for eligible child contacts of < 25 kg. This formulation is procured and provided by the CaP TB project, as the NTP has not yet recommended this regimen, but has approved its use in the context of the study. Prescription is based on the body weight dose range as recommended by WHO [32]. The body weight is measured at the TPT initiation visit and at the TPT outcome visit for both models, and in the facility-based model, it is measured at every follow-up visit. For children of 25 kg or more, the adult RH tablet is be provided. For children receiving an antiretroviral treatment with protease inhibitors (as lopinavir/ritonavir), nevirapine or dolutegravir, 6H is used to avoid the drug-drug interaction between R and these antiretrovirals. Along with the TPT, 10 mg of daily pyridoxine (vitamin B6) is given to each child to prevent peripheral neuropathy.

### Study procedures

#### Symptom screening

Both models use the following symptoms [33] to assess if the contact child has presumptive TB or not:

- Persistent non-remittent cough > 2 weeks
- Reported persistent fever > 10 days
- Reduced playfulness/lethargy/fatigue
- Wheezing > 2 weeks
- Night sweats > 2 weeks
- Documented or reported weight loss, loss of appetite, or no weight gain (failure to thrive) in the last month
- Malnourishment using Mid-Upper Arm Circumference below 125 mm in children 6 months–5 years old

The presence of at least one of these symptoms requires TB investigations at the health facility.

For HIV-positive children, symptoms of any duration are suggestive of TB and the child is immediately referred to the clinic. HIV testing is proposed for 5–14-year-old children with unknown status using two rapid tests, as per national HIV testing guidance. In the community model, the first test is done in the household and if positive, the confirmation test is done at the health facility as per national guidance.

In case a child presents a sign that is not yet suggestive of TB due to its duration (ex: cough for less than 2 weeks), the screening is repeated after 2 weeks’ time.

In addition to the screening for TB symptoms, CHWs have been trained to identify critical signs for urgent referral in case the child needs to be seen urgently by a clinician. These signs are recommended by the Integrated Management of Childhood Illness Handbook of the WHO [34] and include lethargy or unconsciousness, chest indrawing, difficulty breathing, sunken eyes, drinking poorly or not drinking, seizures, severe wasting, severe pallor, and edema of both feet.

#### Adherence assessment

The adherence is assessed at each follow-up visit in both models of care using specific questions on how many doses were missed in the last 4 days, by counting the number of doses taken reported by the parent/guardian in a TPT treatment card introduced by the study and by verifying empty drug blister packs. Parent/guardian received treatment adherence counseling at TPT initiation and during follow-up based on treatment adherence.

#### Safety assessment

At each follow-up visit, the children are assessed for TPT tolerability by CHW using a standard check list of signs suggestive of hepatitis, peripheral neuropathy, and rash that are classically associated with HR (nausea, loss of appetite, vomiting, jaundice, dizziness, tingling, or burning sensation in the extremities). In every cluster facility, a clinician was trained to act as a safety monitor and examine children with problems of tolerability identified by the CHW. In case of serious adverse events, the safety monitor immediately notifies the event to the country principal investigator who informs the sponsor and the ethics committee of the respective country. All adverse events and serious adverse events are coded using the Medical Dictionary for Regulatory Activities (MedDRA) dictionary (version 22.1, September 2019). On a 6-month basis, a safety data review is done by the study management team that is then reported to the sponsor and scientific advisory committee.

### Sample size calculation

For the sample size calculation, we used an estimated 60% completion rate among the eligible children in the facility-based arm based on a recent systematic review [35] and a 10% difference in the community-based arm, considered to be the minimal clinically relevant difference. We considered a cluster coefficient variability of 50% based on the variation in the number of bacteriologically confirmed index cases between the 20 clusters in the year prior to the intervention. An intra-cluster correlation of 0.01 was used. With these parameters, we would need to include at least 1500 declared child contacts by the index case who would be eligible to the TPT to have a power of 85%. With a minimum of 1500 enrolled child contacts, we could maintain at least 80% of power to detect a difference of 10% in the primary outcome between the two arms assuming the proportion with the primary outcome in the control arm ranges from 60 to 70% and of the cluster coefficient variability varies from 50 to 70%. The type I error rate *α* is conventionally fixed at 0.05%. Based on national household statistics per country [36–38], we make the hypothesis of one child under 5 years per household. Looking at the index case TB registrations in the year prior to the intervention, we estimate that it would be possible to include 1500 contact children in a 15-month period. Research assistants in each cluster supervised that all bacteriologically confirmed index cases registered in the NTP treatment register were screened for study eligibility to achieve adequate participant enrolment to reach sample size.

### Data collection

Mixed methods of data collection, quantitative and qualitative, are used. There were no specific plans to promote participant retention to avoid biasing the trial outcomes that the cascade of cares for contact screening and management. The study reimburses participant’s transportation in case for safety reasons only.

#### Quantitative data

Other than the facility registers, study-specific source documents are used. The data for the primary and secondary objectives are collected by the TB focal person in the health facilities and community health workers in the community. There is one research assistant assigned to each cluster health facility who enters data onto tablets using the Research Electronic Data Capture (REDCap) mobile application version 4.9.1, 6 February 2020. Patients’ cost data are collected by research assistants in the REDCap mobile application using an adapted version of the patient cost tool developed by the WHO [39]. At the health system level, data are collected through literature, source documents from the Ministry of Health, primary expenditure analysis, and procurement records by the cost analysis researchers.

#### Qualitative data

The data for the qualitative assessment is collected by the social researchers through in-depth interviews and focus group discussions in English, French, or local language with the help of a local qualitative research assistant. Participants’ confidentiality and privacy are respected throughout the study. During the baseline phase of the study, a qualitative assessment of social determinants has been performed to identify the perceptions of TB, prevention for child contacts, and obstacles for treatment, acceptability, and feasibility of the proposed intervention. Focus group discussions have been organized with TB patients and in-depth interviews have been conducted with health staff, facility managers, CHW, and community leaders. During the implementation phase, data on concurrent acceptability is assessed through periodic supervision meetings of the CHW. A second qualitative assessment will be performed at the end of the intervention focusing on the acceptability and lessons learned. These activities are planned in both models of care implemented in the study.

#### Process data

In the baseline phase, sites were assessed in terms of quality of data collection in the registers and specific practices that would require adjustments for study organization of those sites. During the implementation phase, recruitment logs are filled in by research assistants to document study screening and enrolment process with reasons of refusal.

### Data management

A central data manager coordinates with local data managers to ensure the data entry and verification according to a Data Management Plan. There are three levels of data checking and quality control: at data entry using restricted value set or compulsory fields, at country-level data management running weekly checks, and at central-level data management with a monthly consistency data check. The collected data is anonymized by the use of unique study identification numbers and followed by the investigators through a dashboard system developed at the central level. The tablets used for the study are password-protected and have an individual identification for each research assistant. The tablet data is encrypted when sent to the server. The study database is on a web-based platform provided by REDCap [40, 41], protected by password, encrypted, and hosted at the Institut de Recherche pour le Development in Montpellier, France. The back-up of the database is done on a daily basis on the server of the Institut de Recherche pour le Development in Montpellier.

### Quality management

Each country research team is composed of one study coordinator, one clinical research assistant, and 8 research assistants. All staff is trained on good clinical practices, protocol, and study standard operating procedures. Training of the country research teams took place before the baseline phase and was done by the central research team. CHW were selected based on criteria regarding their education, experience with community activities, and acceptability by the community and capacity for study activities. The site teams (TB focal person, TB nurse, CHW, and safety monitor) were trained before the intervention phase by the country research teams. Each facility cluster has a clinician safety monitor trained to consult children with tolerability complaints and report adverse events. Standard operating procedures, country-specific manuals of procedures (to take into account the implementation specificities of each country), and a quality management plan were developed by the central research team. Clinical research assistants perform internal data monitoring from the eCRF against source documents on a monthly basis, and central site monitoring is done every 3–4 months. In addition, the sponsor performs a yearly site monitoring.

The study is overseen by a steering committee involving all investigators including representatives of the national TB program with monthly calls to discuss study inclusions, challenges, and decisions on study implementation and a scientific advisory committee composed by experts in the field of pediatric tuberculosis and randomized controlled trials with meeting twice a year to discuss study progress, challenges, and safety review. A country community advisory board is constituted to give guidance on the implementation of the community activities and support the study team on patient information and communication.

### Statistical analysis

#### Primary analysis

The denominator for the analysis of the primary endpoint is the number of child contacts < 5 years and HIV-infected 5–14 years declared by the index case at the facility during the inclusion visit. Since discrepancies can be expected between what is declared by the index case and what is observed during contact screening, a sensitivity analysis will be performed using as denominator the number of children < 5 years of age and HIV-infected children of 5–14 years of age identified during the screening. An additional sensitivity analysis will be performed including only participants that followed all study procedures (per-protocol approach) among the declared and then enrolled child contacts. Dropping out of the cascade of cares and potentially being lost to follow-up can be the consequence of the models of care under evaluation. Therefore, lost to follow-up will be kept in the primary outcome analysis. They will be removed from the sensitivity per-protocol analysis.

A generalized linear mixed model with a binomial distribution and logit link function will be used to perform individual-level analysis adjusting for clustering. The regression model will include the fixed effect of treatment assignation and country and one random-effect for the cluster. A degree-of-freedom correction will be applied (between-within method) to deal with the type I error inflation due to the small number of clusters. The primary analysis will focus on the difference between the two study arms adjusted for country, and a secondary analysis will add an adjustment for unbalanced factors (urban/rural, district size) identified in the baseline assessment. For the analysis of the secondary outcomes, a similar mixed model will be used with the same random effects and correction method, focusing on each endpoint of the cascade of care for initiation and completion of TPT and each endpoint of the cascade of care for TB detection. The same model will be applied for the sensitivity analyses.

The proportion of children notified in the facility TPT register among all notified cases during the intervention period will be compared between the two models of care and will also be compared with the same proportions before intervention for the same time period (data collected during the baseline assessment). The proportion of confirmed TB among all pediatric notified cases and the proportion of treatment completion will also be compared between the two models and with the pre-intervention period.

#### Qualitative assessment analysis

All transcripts from the in-depth interviews and focus group discussions are transcribed in French if the activity was conducted in French and in English if the activity was conducted in the local language or English. For the analysis, an interim analysis process will be used. Major themes from the interviews and focus groups will be listed according to the objectives of the study before starting the analyses (a priori codes) and will be enriched if other themes will be found to be relevant to the study objectives (inductive codes). The analysis will be done using the software ATLAS.ti 8 2017.

#### Cost-effectiveness analysis

The two models of care will be analyzed and their cost-effectiveness in each country assessed.

The analysis will be from the healthcare system’s and the primary analysis will generate an incremental cost per Disability-Adjusted Life Year averted for the intervention model of care vs the standard of care, with a mathematical model used to extrapolate effects observed in the trial to a lifetime time horizon. Additional analyses will include reporting of patient costs incurred during illness and care-seeking, and an asset-based wealth quintile of participants, and generation of additional measures of health impact (deaths and TB cases averted).

### Ethical aspects

#### Protocol approval

The study protocol has been submitted and approved by two central Institutional Review Boards (IRBs): Advarra IRB from the USA, which is the sponsor’s institutional IRB, and WHO Ethics Research Committee. In addition, the protocol was submitted and approved by the local IRBs: Cameroon National Ethics Committee for Human Health Research and Research Ethics Committee of the Mbarara University of Science and Technology in Uganda. In Cameroon, it has also been approved by the Direction for Operational Research from the Ministry of Health and in Uganda by the Ugandan National Council for Science and Technology. Any change in the protocol or to the informed consent form that affects the scientific questions and study design or may affect a subject’s willingness to continue participation in the study were considered as amendment and are submitted to all previously described ethics committees after approval from the scientific committee and the sponsor.

#### Informed consent

All consent forms used for the CONTACT study have previously been approved by the central and local ethics committees. Written informed consent is obtained from index cases and contacts, who are informed of the study objectives, procedures, and their risks and benefits. In addition, children older than 7 years in Cameroon and 8 years in Uganda provide written informed assent. Participants with incapacity consent through their legal representative and illiterate participants consent through a witness who is not part of study staff. Country-specific informed consent forms are developed to allow for different standard of care specificities. For index cases, the TB focal person collects the informed consent at the inclusion visit. For contacts in the facility-based model, the TB focal person, assisted by the research assistant, collects the informed consent. In the community-based model, only the research assistant can collect the informed consent. Consent for HIV testing is included in the study consent form.

Individual consent is obtained by the researchers during qualitative activities: focus group discussions and in-depth interviews and also during the patient cost collection for the cost-effectiveness analysis.

#### Handling withdrawals

At any moment, a contact case can withdraw their consent without any consequence for their care and their data prior to the date of withdrawal are kept for analysis. Their case management continues under the NTP guidelines.

#### Confidentiality

Each participant has a unique study code. No directly identifying data is entered into the database. An identification log allows the research assistants to make the link between the code and the name if needed and this log is kept separately in locked study cabinets on site.

### Dissemination

The trial results will be published in peer-reviewed medical journals, preferably open access or guarantying an open access according to international guidelines for authorship. After approval by the scientific committee, the final trial report will be sent to the sponsor, Unitaid, the World Health Organization, and NTP officials.

## Discussion

The CONTACT study has several strengths and limitations.

### Strengths

Methodologically, the use of a randomized cluster-controlled design ensures a good level of evidence. The inter-cluster variability is taken into consideration by the use of a covariate-constrained randomization of the number of index cases per cluster. In addition, the study is using a comprehensive mixed-methods approach that looks at the study goal from different perspectives: quantitative, qualitative, and cost-effectiveness.

The intervention package was conceptualized in a very pragmatic and realistic manner after discussion with end-users, national TB program, and community representative to ensure that it could be implemented by the NTP at the end of the research period. In addition, the intervention is evaluated in two countries with similar TB burden, but very different health system organization and level of community engagement that increases the representativeness of the study results. In Cameroon, the national system is very centralized whereas in Uganda lower level health facilities are capacitated to do TB diagnosis and follow-up. In Uganda, over the last 2 decades, there have been several HIV-related interventions, many of which have been implemented in the communities. The population is used to community activities and a system of CHW is in place (called Village Health Teams) and articulated by the Ministry of Health. The package proposed by the CONTACT study has been inspired by the HIV and malaria community activities [42, 43] and integrates very well in the Ugandan context. Finally, all cluster facilities are supported by the CaP TB program for TB diagnosis and treatment, which reduces the risk of heterogeneity between the clusters and TB detection endpoint assessment bias.

The study is constructed on the framework already existent in the health facilities and uses the health personnel of these health facilities. The main strength is represented by their training and experience in working with TB, and the fact that they are already integrated in the national system, no study additional staff was hired for this purpose. The TB focal person and safety monitor receive an incentive for filling study-specific documentation that is outside their usual work.

### Limitations

Because the cluster sites were limited to the facilities supported by the CaP TB project, it was not possible to select more than 20 clusters. It was impossible to avoid urban facilities, which increases the inter-cluster variability and may increase the risk of cluster contamination due to the more complex system of patients’ reference in cities as compared to rural settings. The proportion of urban clusters is higher in Cameroon as the two selected regions where the CaP TB project takes place include the two biggest cities of the country. In Uganda, some clusters comprise two health facilities to allow for the necessary recruitment capacity and this operational limitation may introduce more heterogeneity in the measurement of the outcomes.

Another limitation is the reliability of source documents from facility registers as compared to study-specific source documents, which can induce an information bias and risk of missing data. To minimize this limitation, a register data quality check was done during the baseline period and in sites where inconsistencies were found, a training on data collection was recommended.

The training of facility personnel on the study procedures, the reinforcement of the study source documents, and the presence of research assistants is likely to increase the quality of the facility-based as compared to routine conditions and may have an effect on the expected difference of primary endpoint between the community-based and the facility-based models. Also, because the duration of the TPT is known to influence the completion of the TPT, which is part of the primary endpoint measure and because NTP was expected to change their guidelines in the coming months, we introduced the 3-month regimen in the facility-based model as well. Therefore, the study standard of care does not fully represent the current standard of care used in both countries. The choice of a very operational and pragmatic adherence measure likely to be well accepted by NTP (recording of the dose intake on a treatment card) relies on the parent/guardian’s understanding and reliability in recording the dose intake and could potentially introduce information bias or desirability bias. To prevent this risk, the CHW and research assistants are asked to systematically reconcile what is recorded by the parent/guardian on the treatment card with the pills remaining in the blisters. In the community-based model, two extra visits after 1 and 2 weeks after starting TPT were requested by the NTP to ensure that no child with TB disease was missed by the symptom screening done by CHW and to verify the tolerability. This results in more frequent assessments of treatment adherence and tolerability as compared to the monthly follow-up in the facility-based model and could introduce an observation bias that could affect the comparison of adherence and safety between the two models.

### Challenges

This is an implementation research which is highly dependent on the health system policy and organization. One of the many challenges to be taken into account is the change of the country guidelines during the implementation of the protocol as these changes require most of the time a protocol amendment with implications on study procedures and organization. Other challenges are related to shortages of TB medication, stock-outs of Xpert MTB/RIF cartridges that affect the identification of bacteriologically confirmed index cases at facility level and staff availability or turn over. The 3RH and 6H TPT drugs were provided by the study to prevent the risk of shortage. One cluster in Cameroon had to be changed after approval of the study protocol due to an unanticipated concurrent community-based intervention that could bias the study outcome measure. Additionally, in both countries, following the request from the NTP that TPT should be initiated by a nurse, the implementation of the intervention package could not rely on CHW only as initially planned and has to involve facility nurses moving to patients’ household. The same applies to HIV testing that cannot be done by trained CHW in Cameroon.

Constant communication with the CaP TB program team and the NTP team at higher and lower levels is crucial to anticipate any operational issue and find solutions to ensure the continuity of study activities according to the protocol. It also reinforces the level of ownership by the NTP and prepares the future scale-up of the intervention. The cost-effectiveness and qualitative research components focusing on acceptability and potential barriers such as stigma around tuberculosis and its association to HIV bring crucial information for future scale-up.

The CONTACT study will bring new evidence of alternative ways for tuberculosis contact management in a more convenient manner for children and their families with an expected impact on TPT uptake, treatment completion, and increase of case detection.

### Study status

The study completed the first phase and participants’ enrolment started on 14 October 2019. Enrolment is expected to be completed in December 2021. The current study protocol version is version 3.0: 24 June 2019.

## Supplementary Information

**Additional file 1.** List of participating clusters.

## Data Availability

The research team at the IRD (Institut de Recherche pour le Développement) in Montpellier, France, will have access to the final trial dataset from both countries.

## Abbreviations

3RH: 3 months of rifampicin and isoniazid daily
6H: 6 months of isoniazid daily
CaP TB: Catalizing Pediatric TB Innovations
CHW: Community health worker
CONTACT (study acronym): Community Intervention for Tuberculosis Active Contact Tracing and Preventive Therapy
CXR: Chest radiography
EGPAF: Elizabeth Glaser Pediatric AIDS Foundation
FDC: Fixed-dose combination
H: Isoniazid
HIV: Human immunodeficiency virus
IRB: Institutional Review Board
MedDRA: Medical Dictionary for Regulatory Activities
MDR TB: Multidrug-resistant TB
NTP: National Tuberculosis Program
R: Rifampicin
REDCap: Research Electronic Data Capture
TB: Tuberculosis
TB-LAMP: TB loop-mediated isothermal amplification
TPT: Tuberculosis preventive therapy
TST: Tuberculin skin test
WHO: World Health Organization
