# Supplementary material for: Community intervention for child tuberculosis active contact investigation and management: study protocol for a parallel cluster randomized controlled trial
Source: Trials. 2021 Mar 2;22:180. doi: 10.1186/s13063-021-05124-9 (PMC7927252; doi:10.1186/s13063-021-05124-9)
Supplement: Supplementary file 1 — Additional file 1. List of participating clusters. [file 13063_2021_5124_MOESM1_ESM.docx]

| **No** | **Facility Name** | **District** | **Population (2016)** | **Adult TPB+** | **Level of facility** | **Urban/rural** |
| --- | --- | --- | --- | --- | --- | --- |
| **Cameroon** | | | | | | |
| 1 | HD Log-Baba | Logbaba | 213 224 | 123 | District Hospital | Urban |
| 2 | HD Mbalmayo | Mbalmayo | 156 597 | 121 | District Hospital | Semi-Urban |
| 3 | HD Bonassama | Bonassama | 337 007 | 106 | District Hospital | Urban |
| 4 | CMA Delangue | Edea | 154 244 | 94 | CMA | Urban |
| 5 | HD Okola | Okola | 69 194 | 90 | District Hospital | Semi-Urban |
| 6 | HD New Bell | New Bell | 860 913 | 177 | District Hospital | Urban |
| 7 | St Jean de Malte | Njombe Penja | 42 749 | 69 | District Hospital | Rural |
| 8 | HR Nkongsamba | Nkongsamba | 160 880 | 61 | Regional Hospital | Urban |
| 9 | HD Mfou | Mfou | 72 235 | 61 | District Hospital | Semi-Urban |
| 10 | HD Yoko | Yoko | 33 519 | 53 | District Hospital | Rural |
| **Uganda** | | | | | | |
| 1 | Ishongororo HC IV | Ibanda | 249,625 | 71 | Health Center IV | Rural |
| 2 | Ruhoko HC IV | Ibanda | 249,625 | 95 | Health Center IV | Rural |
| 3 | Bubaare HC III +  Bwizibwera HC IV | Mbarara | 472,629 | 50 | Health Center IV  Health Center III | Rural |
| 4 | Mbarara Municipal Council HC IV +  Kakoba HC III | Mbarara | 472,629 (195,160^a^) | 60 | Health Center IV  Health Center III | Urban |
| 5 | Bwongyera HC III +  Rwashamaire HC IV | Ntungamo | 483,841 | 59 | Health Center IV  Health Center III | Rural |
| 6 | Itojo Hospital | Ntungamo | 483,841 | 50 | District Hospital | Rural |
| 7 | Ntungamo Ngoma HC III +  Rubaare HC IV | Ntungamo | 483,841 | 50 | Health Center IV  Health Center III | Rural |
| 8 | Kitwe HC IV | Ntungamo | 483,841 | 75 | Health Center IV | Rural |
| 9 | Kitagata Hospital | Sheema | 207,343 | 70 | District Hospital | Rural |
| 10 | Kabwohe Clinical Research Center HC II  + Kabwohe HC IV | Sheema | 207,343 | 50 | Health Center IV  Health Center II | Urban |
